# A cluster randomized controlled trial of an online psychoeducational intervention for people with a family history of depression

**DOI:** 10.1186/s12888-018-1994-2

**Published:** 2019-01-17

**Authors:** Llewellyn Mills, Bettina Meiser, Raghib Ahmad, Peter R. Schofield, Michelle Peate, Charlene Levitan, Lyndal Trevena, Kristine Barlow-Stewart, Timothy Dobbins, Helen Christensen, Kerry A. Sherman, Kate Dunlop, Philip B. Mitchell

**Affiliations:** 1 Psychosocial Research Group, Prince of Wales Clinical School, UNSW, Sydney, Australia; 2 Neuroscience Research Australia, Sydney, Australia; 3 School of Medical Sciences, UNSW, Sydney, Australia; 4 Department of Obstetrics and Oncology, Royal Women’s Hospital, University of Melbourne, Melbourne, VIC 3052 Australia; 5 School of Psychiatry, UNSW, Sydney, Australia; 6 School of Public Health, University of Sydney, Sydney, Australia; 7 Sydney Medical School – Northern, University of Sydney, Sydney, Australia; 8 National Drug and Alcohol Research Centre, UNSW Sydney, Sydney, Australia; 9 Black Dog Institute, Sydney, Australia; 10 Centre for Emotional Health, Department of Psychology, Macquarie University, Sydney, Australia; 11 Centre for Genetics Education NSW Health, Sydney, Australia

**Keywords:** Family history, Major depressive disorder, Bipolar disorder, Online intervention, Psycho-education

## Abstract

**Background:**

People with a family history of major depressive disorder (MDD) or bipolar disorder (BD) report specific psychoeducational needs that are unmet by existing online interventions. This trial aimed to test whether an interactive website for people at familial risk for depression (intervention) would improve intention to adopt, or actual adoption of, depression prevention strategies (primary outcome) and a range of secondary outcome measures.

**Methods:**

In this cluster randomised trial, primary care practises were randomised to either provide the link to the intervention or the control website. Primary health care attendees were invited by letter to opt into this study if they had at least one first-degree relative with MDD or BD and were asked to complete online questionnaires at baseline and 2-week follow-up.

**Results:**

Twenty general practices were a randomized, and 202 eligible patients completed both questionnaires. Thirty-nine (19.3%) of participants were male and 163 (80.7%) female. At follow-up, compared to controls, the intervention group: (i) were more likely to intend to undergo, or to have actually undergone, psychological therapies (OR = 5.83, 95% CI: 1.58–21.47, *p* = .008); (ii) had better knowledge of depression risk factors and prevention strategies (mean difference = 0.47, 95% CI: 0.05–0.88, *p* = .029); and (iii) were more likely to accurately estimate their lifetime risk of developing BD (mean difference = 11.2, 95% CI: -16.52– -5.73, *p* < .001). There were no statistically significant between-group differences in change from baseline to follow up for any of the remaining outcome measures (Patient Health Questionnaire, Perceived Devaluation-Discrimination Questionnaire and Perceived Risk of Developing MDD).

**Conclusion:**

The opt-in nature of the study may have led to participation bias, e.g. underrepresentation of males, and hence may limit generalisability to the broader population at familial risk for depression. This is the first website internationally focusing specifically on informational needs of those at familial risk of depression. Our interactive website can play an important role in improving the outcomes of individuals at familial risk for depression. Testing the intervention in other settings (e.g. psychology, psychiatry, genetic counselling) appears warranted.

**Trial registration:**

The study was prospectively registered with the Australian and New Zealand Clinical Trials Group (Registration no: ACTRN12613000402741).

## Introduction

The strongest risk factor for developing depression is having a history of depression in the family [1]. Adoption, twin, and family studies suggest that major depressive disorder (MDD) and bipolar disorder (BD) are both highly heritable conditions [2] with heritability estimates of around 40% for MDD [1, 3] and 80% for BD [4, 5]. A recent meta-analysis showed that having one first-degree relative (FDR) with MDD more than doubles the risk of developing depression compared with having no FDRs with MDD (OR = 2.1) [6]. With two FDRs the risk is triple that of individuals with no family history of MDD (OR = 3.2) [6]. The same meta-analysis found that having a single FDR with BD increased the risk of developing BD by almost eight times (OR = 7.9) [6].

As a consequence of these relative risk estimates, many individuals with a family history of MDD or BD are frequently concerned about their own and their offspring’s future risk of developing these disorders [7]. Unfortunately they also report that their educational and psychological support needs in relation to their perceived familial risk are largely unmet by currently available psychoeducational resources [8]. The principal need expressed by people at familial risk for MDD or BD is for reliable information, concerning: (i) what causes the disorders to develop; (ii) how to recognise symptoms; (iii) the risk of their current or future offspring developing the disorders; (iv) strategies for reducing risk of developing the disorders; and (v) their individual genetic risk of developing the condition based on family history [8, 9].

Psychiatric genetic counselling is an intervention well suited to meet these needs. It has been found to increase empowerment [10], risk perception accuracy and knowledge [11, 12]; decrease a sense of stigma [12]; and help people with psychiatric disorders and their families to understand the causes of their illness, thus enabling them to adapt more successfully. However, psychiatric genetic counselling is a very new speciality and qualified practitioners are exceedingly rare across the world, and as such innovative approaches are required to meet the needs of people at familial risk for depression and other psychiatric disorders. One very cost-effective strategy that seems well suited to meet the needs of people at familial risk is psycho-education provided through the internet.

The internet is arguably the most efficient way of reaching a large number of individuals across a wide range of geographic and economic settings, and for many is the preferred method of accessing mental health information [8]. It also allows for the delivery of information tailored to the individual user (e.g. personalised risk assessments). Websites that improve depression literacy and/or deliver online therapeutic techniques have been shown to be effective at reducing the symptoms of depression [13–16]. There are several high-quality websites available in Australia that fulfil this function (e.g. www.beyondblue.org.au, www.bluepages.anu.edu.au, https://moodgym.anu.edu.au); however none are targeted specifically at those with a family history of depression.

The information most desired by individuals with a family history of MDD or BD relates to strategies that they or their offspring might use to reduce the risk of onset of depression [8, 9]. There is strong evidence from meta-analyses that prevention trials can be very effective at reducing the likelihood of occurrence of new cases of depression [17, 18]. For those with a familial risk of developing depression, psychological therapy, especially cognitive behavioural therapy, has been shown to be particularly effective at preventing depression [17, 19–22], almost halving the risk of its development in at-risk groups [17, 21–24]. Other factors have been shown to reduce the risk of developing depression, including: (i) regular physical activity [25]; (ii) minimising intake of alcohol (in heavy drinkers) and street drugs [26]; (iii) adequate amounts of sleep [27]; (iv) a ‘Mediterranean’ diet [28]; (v) adequate amounts of vitamin D [29]; (vi) good social support [30]; (vii) being optimistic [31]; and (vii) being religious and/or spiritual [32, 33]. Though the evidence for these strategies is not as strong as for psychological therapies and regular exercise, all have been shown to be associated with a reduced risk of developing depression in at least one study, albeit ranging in terms of methodological rigour. It is vital that at-risk individuals have an easy and effective way of accessing this information to help them decide which strategies to adopt in order to reduce their risk of developing depression.

Online psychoeducational interventions have also proven effective at reducing the stigma surrounding depression [34], and there is evidence that having a genetic explanation for depression can reduce the perceived stigma surrounding the disorder [35]. By providing information about the familial basis for depression, an online psychoeducational tailored intervention may help reduce perceived stigma and encourage those with a family history of depression to adopt strategies for reducing depression risk.

Individuals with a family history of depression have specific psychoeducational needs that are not currently met by existing websites. Given the proven efficacy of online psychoeducational interventions and their potential for reaching a large number of individuals, the benefits of developing a website targeted specifically at those with a family history of depression could be substantial. The primary health care setting is perhaps the most pertinent setting to reach those with a family history of MDD or BD to meet their psychoeducational needs, given the high prevalence of these conditions in patients attending primary care physicians; the prevalence of MDD has been found to be as high as 13.9% and that of BD 1.9% in one study conducted in the primary care setting [36]. Therefore, this study aimed to evaluate, in the primary care setting, whether a novel interactive online psychoeducational intervention targeted at individuals with a family history of MDD of BD was more effective than written information alone at: (i) increasing intention to adopt or actual adoption of strategies to reduce risk of developing depression (primary outcome variable); (ii) lowering levels of depression symptoms; (iii) lowering perceived stigma surrounding mental illness; (iv) improving knowledge of genetic and environmental risk factors for depression; and (v) improving the accuracy in estimating risk of developing depression.

## Methods

### Design

**Fig. 1:**
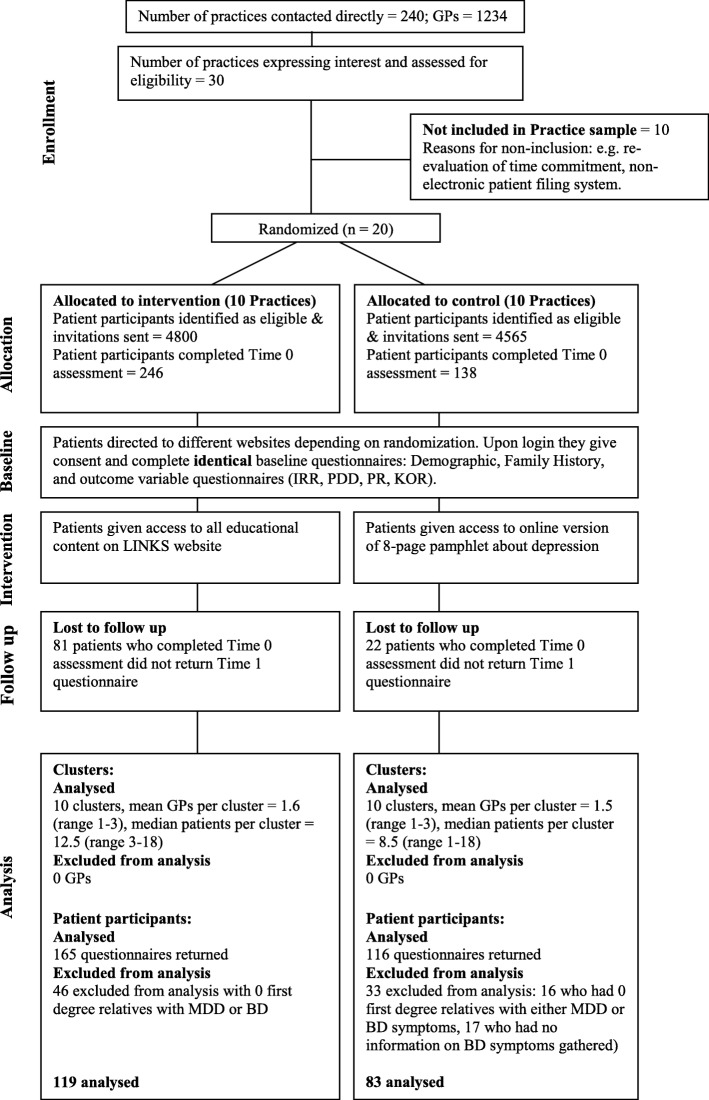
Diagram showing the flow of clusters and participants throughout the study

This cluster randomised controlled trial took place in a general practice (primary health care) setting in Sydney, Australia. General practitioners (GPs) are routinely the first health professional contacted by individuals seeking help for mood disorders, with over 90% of Australians visiting a GP at least annually [37]. Thus general practices were deemed a suitable setting in which to access those at risk of developing depression. Care in these practices is often provided by several GPs. The study design is presented in Fig. 1. The study used a cluster randomised trial design. Randomisation to either the control or intervention condition took place at the general practice level, rather than the individual patient or the individual GP level, so as to optimise use of the intervention and to reduce possible sources of contamination (e.g. via communication between participants in different conditions attending the same practice, communication between GPs in the same practice assigned to different conditions, or via differing approaches of individual doctors to patients assigned to different conditions).

### Sample size

The desired sample size for the study was 240 participants in 20 clusters, randomizing ten clusters each in the intervention and the control arm, with an average of 12 participants per cluster. These sample and cluster sizes were based on an assumed intra-cluster correlation coefficient (ICC) of 0.06 for patients within the same practice, consistent with ICCs observed for psychological variables in GP studies [37]. This would yield 80% power to detect a difference of 22% in the proportion intending to undertake risk-reducing strategies from a baseline proportion of 50% [38]. This proportion corresponds to a medium effect size, the minimum deemed to be of public health and clinical significance. To achieve the desired average per-practice sample size, allowing for attrition, up to 500 invitations per practice were sent (10,000 in total), based on the assumption that 20% of all individuals have at least one FDR with either MDD or BD, and that, of these, approximately 15% would opt-into the trial [39].

### Participants

#### Recruitment of GP practices

GP practices selected for invitation to participate were chosen from across greater Sydney to be as geographically and socioeconomically diverse as possible. Practices were identified using an existing GP database. GPs were sent letters of invitation and were offered a AUD$1500 reimbursement per practice and 40 Continuing Medical Education points for each participating GP in the practice as an acknowledgement of the time commitments required to participate in the study. Those practices/GPs who returned an expression of interest form were contacted to arrange a meeting to discuss the study. The research team provided personalized academic detailing, which included an explanation of the study materials and obtained written consent from individual GPs within the practice to participate.

#### Recruitment of patients

Patients were eligible for inclusion in the study if they: (i) had a family history of at least one FDR with MDD or BD; (ii) were able to give informed consent; (iii) were proficient readers in English; (iv) were aged between 18 and 75 years, and (v) had attended the practice in the past 2 years. As many GPs do not elicit and systematically record a family history of depression, patients were asked to self-identify as having a FDR with MDD or BD. Both individuals who had experienced an episode of MDD or BD, and those who had not, were eligible to participate.

## Materials and measures

### Interventions

#### Intervention

A psychoeducational website (*LINKS* http://links.neura.edu.au) targeting people with a family history of depression was developed based on the findings from a previous study on the educational needs of individuals with a family history of depression [8] and in consultation with a multidisciplinary committee with expertise in psychiatry, psychiatric genetics, psychology, general practice, e-medicine in the psychiatry setting, genetic counselling, and genetics education. The website includes information on both the risk factors associated with developing depression (with information on the likelihood of depression occurring, early signs and symptoms of depression, environmental risk factors such as stress and lifestyle, genetic risk factors) and evidence-based strategies for reducing risk of developing depression (psychological therapy, regular physical activity, getting adequate amount of sleep, Mediterranean-style diet, minimising alcohol and street-drug use, good social support, optimism/having a positive attitude, and religiosity/spirituality). The information was conveyed via 50 screens and eight embedded videos, with an emphasis on visual illustrations of key concepts over text, e.g. ‘The mental illness jar model’ developed by Peay and Austin [40]. Where text was used, it was at a ninth-grade reading level. A risk assessment tool was also included in the website. Upon completing a family history of MDD and bipolar disorder questionnaire, the Family History Screen (FHS) [41], the user received a visualised estimate of lifetime risks and risks over the next year of developing MDD or BD relative to the general population. In total 12 different risk scenarios could be visualised. The features of the website are described in more detail in a previous paper [42].

#### Control condition

The control condition was a link to an online version of a leaflet on depression produced by *beyondblue* (the Australian national depression initiative), which briefly described depressive symptoms, strategies for reducing depression, and where to seek treatment.

### Questionnaires

Participants completed two measures at baseline only (the FHS and items eliciting socio-demographic variables) and an additional five measures at both baseline and 2-week follow-up. These measures are described in Table 1.

**Table 1:** Description of measures and time points administered

Measure	Description of Measure	Baseline*	2-week follow-up
Socio-demographic Questionnaire	Measures sex, age, education level, employment status, marital status, country of birth and language spoken at home.	✓	
Family History Screen (FHS) [41]	Screens lifetime history of MDD and BD symptoms of the participant and of all first-degree relatives. Lifetime history is based on self-assessment rather than by clinician diagnosis.	✓	
Intention to adopt, and adoption of, risk reducing strategies (IRR)	8-item scale measuring whether participants intended to or had already: (i) undergone psychological therapy, (ii) been taking regular, moderate-intensity exercise, (iii) been sleeping 7-9 hours per day, (iv) started a low-fat, ‘Mediterranean’ diet, (v) been taking vitamin D, (vi) been ensuring they had sufficient social support, (vii) been trying to adopt optimistic or positive mental attitude, (viii) been developing their spirituality. 5-point response option: 0–Do not intend to in the next 6 months; 1–No, but intend to in the next six months; 2–No, but intend to in the next 30 days; 3–Have already for < 6 months; 4–have for > 6 months”. The 5 items were collapsed into a single dichotomous variable: (0)–no intention to adopt the risk-reducing strategy vs (1,2,3,4)–intend to or have already adopted the risk-reducing strategy.	✓	✓
Patient Health Questionnaire (PHQ9) [54]	10-item self-report scale assessing symptoms based on DSM-IV criteria for MDD. 4-point response scale. Max. score = 30. Higher score = more depressive symptoms.	✓	✓
Perceived Devaluation-Discrimination Questionnaire (PDD) [55]	12-item scale measuring perceived social stigma surrounding mental illness. 4-point response scale. Max. score 48. Higher score = more perceived stigma.	✓	✓
Knowledge of Risk Factors and Risk-Reduction Strategies for Depression (KOR)	10-item veridical test, developed for this study, designed to assess knowledge of proven risk factors for developing depression and strategies to reduce risk of developing depression. True/False answer. Max. score = 10. Higher score = more accurate knowledge.	✓	✓
Perceived Risk of Developing Depression and Bipolar Disorder (PR) [56]	4-item scale adapted from a previous study [56]. Two items each pertaining to MDD and BD. The first of these two items measures perceived relative risk of developing the disorder sometime in the future compared to others of the same age and gender (5-point scale; Max. score = 5, higher score = higher perceived risk). In second of two items respondent indicate their perceived % chance of developing the disorder sometime in the future (0-100 VA scale; Max. score = 100; higher score = higher perceived risk)	✓	✓

### Procedures

Each practice was randomised once signed consent was obtained from the participating GPs. The 20 participating practices were randomly assigned based on anonymous practice ID numbers to either the intervention or control condition according to a 2 × 10 block design generated by an online randomisation tool (https://www.random.org/). Following randomisation, practice managers were instructed by researchers on how to implement the study. Researchers worked with staff at GP practices to generate random lists of up to 500 patients per practice. GPs were asked to scan these lists and exclude those patients who would not be suitable (e.g. cognitive impairment, severe illness, patients with a psychiatric disorder that was currently not well controlled). Those deemed suitable were sent a letter of invitation by their GP. Potential participants were blinded to their intervention assignment by being told in the invitation letter that the purpose of the study was to compare two types of educational interventions. The letter invited those who self-identified as having at least one FDR who had an episode of depression at some point in their life to enter the *LINKS* study. The letter contained a link to either the *LINKS* website or to the control website depending on participants’ GP practice allocation. Upon following the provided link, participants were asked to give consent. They were then asked to complete the baseline survey, containing the socio-demographic items, FHS, and outcome measures. After completing the baseline survey, participants were given free access to the website material and were asked to complete the follow up survey within 14 days. Reminders were sent as required.

### Statistical analyses

In order to estimate changes in outcome variables over time while taking account of clustering, random-intercepts hierarchical linear regression models were fitted for each outcome variable, with the second measurement occasion (2-week follow-up) as the outcome variable, intervention group as the primary predictor, baseline score for the same outcome variable as covariate, and practice ID as the random factor. For dichotomous variables, relative odds were estimated using hierarchical logistic regression. Differences between the intervention and control arms in changes in quantitative outcomes were estimated using normal hierarchical linear regression. Intra-cluster correlation coefficients (ICCs) were calculated for all variables. Where the estimated ICC from the hierarchical model was negative for any analysis, it was assumed to be 0, as true negative ICCs are rare in this context. Baseline differences in gender, age, and family history of MDD or BD were examined for possible inclusion as covariates in regression models (discussed in ‘Results’ below). Analyses were performed using the *lme4* [43], *ICC* [44] and base packages in R [45].

### Technical limitations and their impact

Due to a technical error with the automated screening procedure on the *LINKS* website, 79 participants who proceeded through the trial had no FDRs with a history of MDD or BD. These participants were excluded from analyses. A further technical error resulted in the questions relating to symptoms of BD not being present in the FSH questionnaire for the first 22 participants who used the control group’s website; thus these participants could not be included in analysis. In addition, as a result of a technical error involving the website used to administer the follow-up questionnaires, only one of the eight strategies for reducing risk of depression contained in the baseline questionnaire had matching data collected in the follow-up questionnaire: intention to undergo or currently undergoing psychological therapy as a preventative strategy. While this error was regrettable, this risk reduction strategy has the most evidence for efficacy at preventing depression [17, 21–24]. Finally, while the study protocol had included a six-month follow-up [46], financial and time constraints meant this could not be completed.

## Results

Of the 30 practices that expressed an interest in taking part in the trial, ten did not take part, either because they subsequently decided they could not devote sufficient resources to conducting the trial, or because they had paper-based filing systems that were unsuitable for generating patient lists. This left 20 practices (clusters) to take part in the trial (Fig. 1).

The median number of participants per practice whose results were analysed was 11. Although the practices *invited* to participate were selected to be as culturally and socioencomically diverse as possible, of the practices who agreed to take part in the study 12/20 practices were located in suburbs in the top quartile of rankings of the 2011 Socioeconomic Index For Areas (SEIFA) [47], 7/20 in the third quartile, and 1/20 in the second quartile (Table 1). As such SEIFA percentile rank was included in all analyses as a level-2 covariate.

Three hunderd and 84 individuals completed the baseline questionnaire and, of these, 281 also completed the two-week follow-up questionnaire. Excluded from analysis were 62 participants who indicated in the FHS questionnaire that they had no FDRs with a history of MDD or BD symptoms and 17 who did not have information about FDRs with BD symptoms collected, leaving 202 participants in total (control = 83, intervention = 119) included in analyses.

Baseline characteristics at the cluster and individual level are shown in Tables 2 and 3. Of the demographic variables, only family history with symptoms of MDD or BD showed any imbalance between intervention and control at baseline, with 33.6% of participants in the intervention group having at least one FDR with bipolar symptoms, compared to 20.5 in the control group. Consequently family history with BD was included as a level-1 covariate in analyses.

**Table 2:** Baseline information for each trial arm at the practice (cluster) level

Variables		Intervention group (*N* = 10) Mean (SD)	Control group (*N* = 10) Mean (SD)	Total sample (*N* = 20) Mean (SD)
Mean no. of participating GPs per practice/cluster	Range: 1, 3	1.6 (0.8)	1.5 (0.7)	1.55 (0.8)
Mean no. of GPs per practice/cluster (participating and non-participating)	Range 1, 20	7.9 (4.6)	9.7 (7.2)	8.8 (5.9)
	Level	*N* (%)	*N* (%)	*N* (%)
Full-time practice manager	Yes	6 (60)	7 (70)	13 (65)
	No	4 (40)	3 (30)	7 (35)
Billing arrangements^a^	Bulk-billing all patients	2 (20)	5 (50)	7 (35)
	Private Billing	8 (80)	5 (50)	13 (65)
SES of Practice Location^b^	Rank (in Quartiles)			
	75–100	6 (60)	6 (60)	12 (60)
	50–75	3 (40)	4 (40)	7 (35)
	25–50	1 (0)	0 (0)	1 (5)
	1–25	0 (0)	0 (0)	0 (0)

^a^Bulk-billing all patients refers to practices where the cost of the visit for all patients is covered by Medicare, Australia’s universal health care plan. Private billing in this case refers to practices that had either only private billing (i.e. visit paid for upfront or by a private health care fund) or a combination of private billing and bulk-billing

^b^Based on Percentile Ranks within NSW contained in the 2011 Socioeconomic Index for Areas (SEIFA) published by the Australian Bureau of Statistics. Higher ranks indicate higher socioeconomic status

**Table 3:** Demographic and family history variables at the patient (individual) level

Variables		Intervention group (*N* = 119) Mean (SD)	Control group (*N* = 83) Mean (SD)	Total sample (*N* = 202) Mean (SD)
Age	Range: 18, 74	44.0 (14.7)	40.2 (12.4)	42.42 (13.9)
	Level	*N* (%)	*N* (%)	*N* (%)
Gender	Male	25 (21.0)	14 (16.9)	39 (19.3)
	Female	94 (79.0)	69 (83.1)	163 (80.7)
Highest education level achieved	Other	2 (1.7)	2 (2.4)	4 (2.0)
	Some High School	15 (12.6)	9 (3.6)	24 (11.9)
	Graduated High School	8 (6.7)	3 (10.9)	11 (5.4)
	Vocational college	33 (27.7)	24 (28.9)	57 (28.2)
	Degree/Postgraduate degree	61 (51.3)	45 (54.2)	106 (52.5)
Occupation	Wages/Salary/Self-Employed	79 (66.4)	54 (65.1)	133 (65.8)
	Student	10 (8.4)	9 (10.8)	19 (9.4)
	Retired/Unemployed	20 (16.8)	8 (9.6)	28 (13.9)
	Other	10 (8.4)	12 (14.5)	22 (10.9)
Marital status	Married/living as married	50 (42.0)	33 (39.8)	83 (41.1)
	Never married/Divorced/Widowed	69 (58.0)	50 (60.2)	119 (58.9)
Children	Yes	73 (61.3)	43 (51.8)	116 (57.4)
	No	46 (38.7)	40 (48.2)	86 (42.6)
Country of Birth	Australia	96 (80.7)	56 (67.5)	152 (75.3)
	Other	23 (19.3)	27 (32.5)	50 (24.7)
Language spoken mostly at home	English	119 (100.0)	79 (95.2)	198 (98.0)
	Language other than English	0 (0.0)	4 (4.8)	4 (2.0)
Personal symptoms of MDD or BD^a^	MDD Symptoms Only	60 (50.4)	47 (56.6)	107 (53.0)
	BD Symptoms^c^	14 (11.8)	8 (9.6)	22 (10.9)
Symptoms of first-Degree relatives^b^	1 FDR with MDD Symptoms Only	55 (46.2)	35 (42.2)	90 (44.6)
	2 FDR with MDD Symptoms Only	19 (16.0)	24 (28.9)	43 (21.3)
	3 FDR with MDD Symptoms Only	3 (2.5)	7 (8.4)	10 (5.0)
	> 3 FDR with MDD Symptoms Only	2 (1.7)	0 (0.0)	2 (1.0)
	1 FDR with BD Symptoms^c^	33 (27.7)	15 (18.1)	48 (23.8)
	2 FDR with BD Symptoms^c^	7 (5.9)	2 (2.4)	9 (4.5)

^a^As assessed by the Family History Screen

^b^Total number in this analysis was 202 after excluding participants in the control group whose questionnaires were incomplete. Entry refers to type of history (MDD only versus BD) in first-degree relatives

^c^Either with or without presence of MDD symptoms

### Intention to adopt, and adoption of, risk reducing strategies

The increase in the proportion of individuals who intended to undergo or had undergone psychological therapy from baseline to 2-week follow-up was 22.1%, in the intervention group, compared to 0.0% increase in the control group (Table 4). This meant that, after adjusting for baseline, the estimated odds of intending to undergo therapy or currently undergoing therapy were 5.83 times higher in the intervention group (95% CI: 1.58–21.47, *p* = .008).

**Table 4:** Results of outcome variables

Variables	Intervention group			Control group				Adjusted		
	N	Baseline	Follow-Up	N	Baseline	Follow-Up	ICC	Comparative statistic^a^	95% CI statistic	*p*
Intention to Undergo Therapy as a Risk Reduction Strategy	113	0.47 (.50)	0.69 (.46)	83	0.75 (.44)	0.75 (.44)	0^b^	5.83	1.58–21.47	**0**.**008**
Patient Health Questionnaire	119	6.49 (6.0)	5.48 (5.5)	81	7.56 (6.3)	6.64 (5.3)	0.068	0.25	−0.74 – 1.24	0.625
Perceived Devaluation and Discrimination	117	20.62 (4.8)	19.74 (5.1)	83	20.57 (5.4)	19.61 (5.8)	0.044	0.29	−0.98 – 1.56	0.662
Knowledge of Risk Factors	117	6.08 (1.8)	7.16 (1.6)	83	6.08 (1.8)	6.74 (1.5)	0^b^	0.47	0.05–0.88	**0.029**
Perceived Risk										
MDD										
Comparative Risk	114	2.47 (1.0)	2.34 (1.1)	83	2.54 (1.2)	2.40 (1.1)	0^b^	−0.03	−0.27 – 0.20	0.774
% Risk	114	46.93 (24.6)	43.07 (26.4)	83	51.69 (30.6)	49.16 (30.1)	0.026	−2.66	−7.87 – 2.55	0.319
BD										
Comparative Risk	113	1.54 (1.0)	1.51 (1.0)	83	1.43 (1.3)	1.63 (1.3)	0.054	−0.21	−0.51 – 0.09	0.195
% Risk	114	35.44 (22.5)	26.67 (18.8)	92	32.77 (28.2)	35.30 (28.6)	0.002	−11.13	−16.52 – −5.73	**< 0.001**

Significant findings in bold

^**a**^Comparative statistics are: odds ratio for dichotomous variable intention to undergo therapy (0 – no intention to undergo therapy vs 1 – intention to undergo therapy or actually have undergone therapy) and estimated mean difference for all remaining continuous variables

^b^If ICC < 0 it was assumed to be equal to 0. All analyses used randomisation as the primary predictor and: (1) family history with MDD and BD symptoms, and (2) SES of GP practice location, as covariates

### Knowledge of risk factors and risk reduction strategies for depression

The estimated increase in knowledge of depression risk factors was 0.47 points greater in the intervention group than in the control group (95% CI: 0.05–0.88, *p* = .029).

### Perceived risk of developing bipolar disorder

Being allocated to the intervention group also resulted in 11.3% greater accuracy in estimating risk of developing BD compared to the control group (95% CI: -16.52 – -5.73, *p* < .001). That is, intervention group participants were less likely to overestimate lifetime risk for BD (26.67%) compared to control participants (35.50%). The correct lifetime risk for BD was 10%. Perceived lifetime risk for MDD was not significantly different between the intervention group (43.07%) and the control group participants (49.16%), with the correct lifetime risk being 25%. There were no statistically significant between-group differences in change from baseline to follow-up for any of the remaining outcome variables (Patient Health Questionnaire, Perceived Devaluation-Discrimination Questionnaire and Perceived Risk of Developing MDD).

## Discussion

This cluster randomised controlled trial tested a novel interactive online psychoeducational intervention developed specifically for individuals with a family history of MDD and/or BD. The results show that the *LINKS* website increased users’ intention to adopt or actual adoption of psychological therapy as a preventative measure against depression. Psychological therapy is the depression prevention strategy that has by far the most evidence for its efficacy, thus it is encouraging that exposure to the *LINKS* website was associated with an increase in users’ willingness to seek therapy in order to reduce their future risk of developing depression. The website also increased participants’ knowledge of both the risk factors for developing depression and strategies for reducing depression. Psychoeducational interventions have been shown to be associated with compliance with medication [48] and willingness to seek help for depression [49]. Though it was not tested statistically in this study, it seems likely that the increase in knowledge of risk factors and prevention strategies could have been the cause of the observed increase in willingness to seek therapy. Future studies may wish to test the relationship more formally via structural equation modelling.

Importantly, the *LINKS* website also improved participants’ accuracy at estimating the future risk of developing bipolar disorder. Many with a family history of MDD and/or BD greatly overestimate the risk of passing this condition to their children [2, 50] and can be reluctant to start a family as a result [35]. A resource that allows those at familial risk of MDD or BD to develop more realistic estimates of future risk of BD to their children could be very valuable in helping allay the fears of these individuals and assist them in making more informed reproductive decisions. The reduction of overestimation of risk for BD may also lead to decreased self-stigmatisation. Research demonstrated that stigma is experienced and internalised by family members of people with mental illness, causing adverse outcomes including psychological distress and decreased quality of life [51], which may be mitigated by more accurate risk estimation for BD. By contrast, the *LINKS* website failed to reduce the percentage of participants overestimating their risk of MDD; one might speculate that risks for BD may be better retained because of a perception that BD is a more serious disorder than MDD.

The intervention did not produce a significant reduction in self-reported depressive symptoms or perceived stigma surrounding MDD and BD. While psychoeducational websites have been found to be as effective at reducing depressive symptoms as websites that deliver cognitive behavioural therapy [16], there are indications that, even for online cognitive behavioural therapy, a longer time frame is generally required to observe significant improvement in symptoms [52]. Psychoeducational interventions have also proven effective at reducing stigma surrounding mental illness [53], and there is evidence that having a genetic explanation for depression can reduce the perceived stigma surrounding the condition [35]. Given that there was only 2 weeks between baseline and follow-up measures in this study, a significant change in depressive symptoms and perceived stigma was perhaps unlikely.

### Study limitations

The technical difficulties in the study were disappointing. Chief among these was the omission of questions concerning participants’ intentions to engage in the seven other strategies for reducing depression from the follow-up questionnaire. Fortunately, we did obtain data on change in willingness to adopt the preventative factor with by far the most evidence, however the missing questions pertained to lifestyle factors that do not have the potential stigma of psychological therapy and which participants may have been thus more willing to adopt. The other main limitation was the lack of a longer-term follow-up. Health education delivery systems typically take longer periods of time to result in significant behavioural change. It would be interesting to see whether exposure to the website resulted in actual change in uptake of risk reduction strategies, and whether other variables such as depression symptoms or perceived stigma were affected over a longer time frame. Another limitation worth mentioning is that web analytic data was not collected on how many times or for how long each participant visited the *LINKS* website during the 2-week study period. Inclusion in the analysis of number of site visits and/or duration of the average visit as level-1 covariates may have provided a more detailed picture of the relationship between exposure to the site and the outcome variables. Another limitation is that only 19% of participants were male and hence generalisability of the findings to men may be limited. Self-assessment of diagnosis of MDD or BD in participants’ FDRs is also a limitation. Final limitations relate to the lack of data on the impact of family history, about the care received and the frequency of GPs’ reports on family history.

### Practical implications

The results of this study highlight the promise of targeted online psychoeducational interventions for delivery of information relevant to those at familial risk of psychiatric conditions. The fact that the website resulted in improved knowledge of risk factors, prevention strategies, and accuracy of estimated future risk should encourage health care providers to invest in the development of similar online psychoeducational resources for other psychiatric conditions. The website is also available for translation into other languages. Although the website was tested on patients in the primary health care setting, it would also be suitable for patients concerned about their familial risk of depression who are seen by psychiatrists, psychologists, genetic counsellors and clinical geneticists. *LINKS* and similar interactive educational websites should be recommended by health professionals for those who are concerned about their familial risk of developing other psychiatric disorders and medical conditions. Another application of the website in the healthcare system might be to screen individuals for early signs and symptoms to target it to subsyndromal individuals as the most likely group to benefit from the education on prevention strategies.

### Research recommendations

Results from this study demonstrate that an interactive, online psychoeducational website can increase both knowledge of strategies to prevent depression and willingness to adopt therapy as a depression prevention strategy in people with a familial risk. It would be worthwhile testing whether the *LINKS* website or similar websites also result in willingness to adopt other lifestyle (e.g. exercise, sleep, diet) or psychosocial (e.g. optimism, social support, or spirituality) prevention strategies in a similar way. In order to test whether the increase in knowledge of, and intention to adopt, risk reduction strategies observed over the 2-week study period translates to actual adoption of those strategies, future studies should examine change over a longer time frame (e.g. 6 months or 1 year). A longer time frame might also allow for manifestation of observable changes in actual depression symptoms or in perceived stigma. It would also be of interest to survey GPs regarding the intervention and assess its impact on patients’ help-seeking behaviours*.* Finally, future large-scale prospective studies should also assess whether the impact of providing information about early warning as part of the intervention may lead to earlier detection and treatment.

## Abbreviations

BD: Bipolar disorder
FDR: First-degree relative
MDD: Major depressive disorder
